# Deep and Machine Learning Using SEM, FTIR, and Texture Analysis to Detect Polysaccharide in Raspberry Powders

**DOI:** 10.3390/s21175823

**Published:** 2021-08-30

**Authors:** Krzysztof Przybył, Krzysztof Koszela, Franciszek Adamski, Katarzyna Samborska, Katarzyna Walkowiak, Mariusz Polarczyk

**Affiliations:** 1 Food Sciences and Nutrition, Department of Food Technology of Plant Origin, Poznan University of Life Sciences, Wojska Polskiego 31, 60-624 Poznan, Poland; krzysztof.przybyl@up.poznan.pl (K.P.); adams.franciszek@wp.pl (F.A.); 2 Department of Biosystems Engineering, Poznan University of Life Sciences, Wojska Polskiego 50, 60-625 Poznan, Poland; 3 Institute of Food Sciences, Warsaw University of Life Sciences WULS-SGGW, Nowoursynowska 159c, 02-787 Warsaw, Poland; katarzyna_samborska@sggw.edu.pl; 4 Food Sciences and Nutrition, Department of Physics and Biophysics, Poznan University of Life Sciences, Wojska Polskiego 28, 60-637 Poznan, Poland; katarzyna.walkowiak@up.poznan.pl; 5 Main Library and Scientific Information Centre, Poznan University of Life Sciences, Witosa 45, 61-693 Poznan, Poland; mariusz.polarczyk@up.poznan.pl

**Keywords:** raspberry powders, FTIR, SEM, ANN, texture analysis, dehumidified spray-drying

## Abstract

In the paper, an attempt was made to use methods of artificial neural networks (ANN) and Fourier transform infrared spectroscopy (FTIR) to identify raspberry powders that are different from each other in terms of the amount and the type of polysaccharide. Spectra in the absorbance function (FTIR) were prepared as well as training sets, taking into account the structure of microparticles acquired from microscopic images with Scanning Electron Microscopy (SEM). In addition to the above, Multi-Layer Perceptron Networks (MLPNs) with a set of texture descriptors (machine learning) and Convolution Neural Network (CNN) with bitmap (deep learning) were devised, which is an innovative attitude to solving this issue. The aim of the paper was to create MLPN and CNN neural models, which are characterized by a high efficiency of classification. It translates into recognizing microparticles (obtaining their homogeneity) of raspberry powders on the basis of the texture of the image pixel.

## 1. Introduction

Raspberries constitute a rich source of bioactive compounds. Red raspberries (*Rubus idaeus* L.) include diverse nutritious components and phytochemical constituents, which are important in leading a healthy lifestyle [1]. It is highly recommended to consume raspberries on account of their nutritious components [2]. It was found that a diet based on eating fruits and vegetables has influence on lowering or reducing, among other things, diabetes, hypertension, or body weight (in people who are overweight) [3,4].

In view of the above, more and more endeavors are being made to find methods that support food preservation, which are aimed at lengthening the durability of food [5]. One of the popular methods of food processing is spray drying. This technique started to be developed by Samuel Percy in the 1870s [6]. Currently, the above method is used in food products in the form of solutions and suspensions—for example, juices and juice concentrates, for instance strawberry juice concentrates [7], milk [8], and honey [9]. It is worth noting down that also an innovative way of spray drying was used in the paper, which differs from traditional solutions. It should also be added that one of the methods of artificial intelligence in the form of artificial neural networks (ANN), whose functioning is based on using appropriate mathematical or programming formulas, was used in the paper [10,11,12]. Artificial neural networks are most commonly used in terms of data, which do not have simple and organized structure calculations. ANN are used above all in stochastic issues, and the ones related to information processing, among other things, with images (bitmap) [10], acoustic emission [13], and numerical data [14,15]. The networks were also used in the process of deep learning, which includes extended methods of image processing. One should not forget that Convolutional Neural Networks (CNN) [10,16] are enjoying more and more popularity. CNN allows prediction [17,18], classification [19], analysis, data association, signal filtering, and process optimization [20,21] in a much shorter time. In the context of deep learning, special attention should be paid to the package called TensorFlow. It is a set of functions enabling the creation of neural networks in a relatively simple and clear way. It allows carrying out operations on tensors, whose modifications constitute the basis for the functioning of artificial neural networks. Another package based, among other things, on the TensorFlow module is the package called Keras. Keras offers solutions that substantially shorten records of artificial neural network in the form of source code. Functions and methods of the Keras package allow preparing input data for a neural network, for example such as image conversion to tensor or the other way round, in an efficient way [13]. In the research, electron microscopy was carried out (SEM) [22], which is currently gaining popularity in terms of recognizing the morphological structure of food. The authors noticed that the morphological structure of fruit powder particles allows an efficient evaluation of their quality [4,7,12,23]. Determining texture parameters was based on a well-known matrix of co-occurrence of gray bands (GLCM) isolated from scanning images [7,24,25,26]. It should be added that also infrared spectroscopy, which is a fast and non-invasive method providing high effectiveness of identifying food products, is attracting more and more interest [27]. It was found that this method turned out to be more effective than the existing solutions up to date [28]. It is proven by a wide range of physiochemical markers for fruit powders. Those analyses are expensive and time-consuming, which eliminates them from application during the controlling process. In the research, evaluation of the effectiveness for Convolution Neural Network, Multi-Layer Perceptron Network, and FTIR was carried out in the process of identifying nine research trials of raspberry powders on the basis of the degree of saccharification and the type of polysaccharide. The utilitarian aim of the research was to create neural models capable of identifying fruit powders obtained in the process of low-temperature spray drying in a quick and non-invasive way [29]. Innovative solutions were based on image technique SEM (having an indirect effect on texture and image pixel) and on spectrum in absorbance function (FTIR).

## 2. Materials and Methods

### 2.1. Material

The research material consisted of raspberry powder samples obtained from raspberry concentrate and carrier (research class). For the needs of the research, the raspberry concentrate with 65°Brix extract was obtained from a 250 kg batch of this product from Białuty Public Limited Company (Błonie, Poland) [4]. Each research sample differed in terms of proportion and type of carrier (Table 1). In the research, the three most commonly present carriers used as food ingredients were used, namely maltodextrin (Amylon, Czech Republic), gum arabic (Colian, Kalisz, Poland), and inulin (Orafti GR, Beneo, Germany). The carrier share was 50, 60, and 70% solids (*w*/*w*), and the concentration of solution was 50% (*w*/*w*). In the research, the solutions were prepared by mixing raspberry concentrate with an appropriate amount of water and polysaccharide, which were then dried in 600 g portions each [4].

The basic physiochemical parameters of powders, which are subject to this research, occur in the following range: moistness 0.6–8.0% [4], water activity 0.17–0.29 [4], bulk density 500–670 kg/m^3^, fluidity expressed with Hauser coefficient 1.23–1.36.

### 2.2. Scaning Electron Microscopy

During the analysis of image textures, digital images acquired from scanning electron microscope (SEM) type XL (Phenom) with the accelerating voltage of 5 kV were used. A total of 215 digital images were taken with a resolution of 1024 × 1024 120dpi, 24-bit color depth, and 120 dpi accuracy, which were saved in the form of the uncompressed format TIFF.

### 2.3. Image Processing

**Figure 1:**
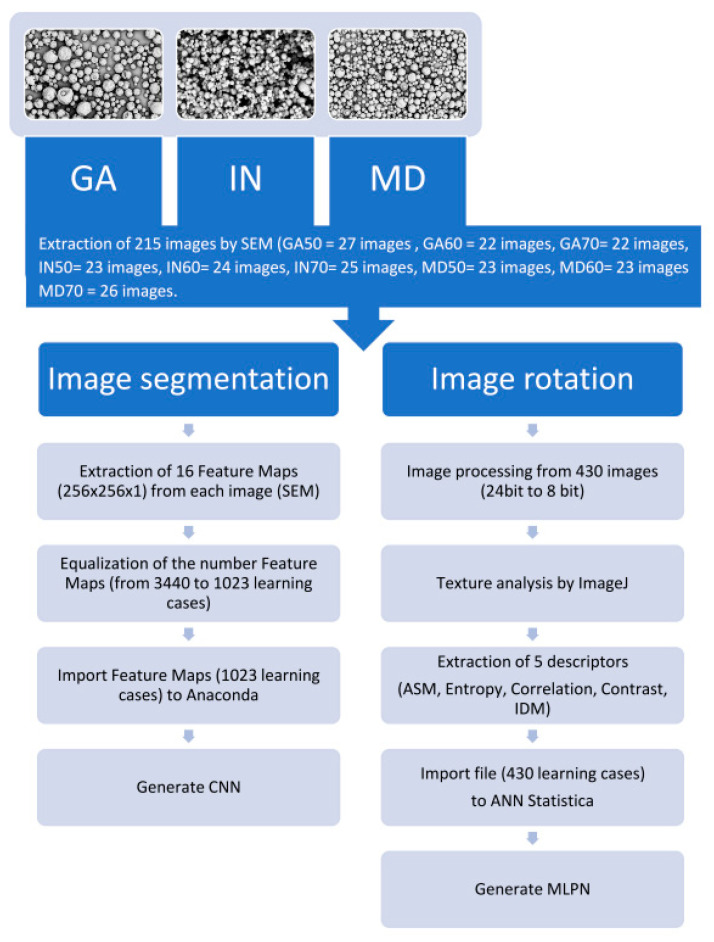
Scheme of image processing.

In the first research variant, point processing of images was carried out in 215 microscopic images. The aim of the operation on microscopic images was to expose structural information of raspberry powders included on a bitmap. In order to carry out this operation, an original software called Przybył Image Detector system (“PID system”) (Poznan, Poland) [30] was applied to rotate 215 digital images by 90 degrees. In the end, 430 microscopic images were obtained (230 scanning images for 0° and for 90°). In the next stage, the analysis of texture was carried out with a gray-level co-occurrence matrix (GLCM) [7,25,31], which—in a three-dimensional graphic—allowed presenting details regarding surface with mathematical function (procedural textures) [15]. In order to do it, digital image—from 24 to 8-bit color depth—processing was carried out with a “PID system”. As a result, digital images were acquired with 1024 × 1024 resolution and 256 shades of grayness in. TIFF format. The prepared base of monochromatic images (8 bit) was imported to the tool called ImageJ (Available online: https://imagej.nih.gov/, accessed on 20 August 2021) [32], with the help of which texture descriptors were properly extracted: Angular Second Moment (ASM), Entropy, Correlation, Contrast, and Inverse Different Moment (IDM) [15,33] (Figure 1).

In the second research variant, image segmentation out of 215 microscopic images was also carried out with a “PID system”. From each digital image with 1024 × 1024 resolution, 16 bitmaps (256 × 256) were isolated. They were saved as new images in. TIFF format, creating in this way a single training case. This operation was repeated, and as a result, the total of 3440 cases was obtained. In the next step, the number of bitmaps was reduced so that each of the research classes had the same number of cases (Figure 1). As a result, the training set for CNN contained 1023 bitmaps (training cases).

### 2.4. The Structure of Neural Network

As part of the research, the process of deep learning and machine learning of networks was carried out. In order to do it, an MLP neural network was designed for the first research variant, for which the training set represented 5 input data related to image texture and 1 output data determining the class of raspberry powders. The aforementioned first research variant included 430 microscopic images determining training cases. The structure of the devised MLPN comprised the following:

- Input layer (InputLayer) i.e., numerical data in the form of 5 texture descriptors;
- Hidden layers, for which the range of layers was set between 10 and 25;
- Nine output neurons in the output layer comprising 9 classes of the research trials of raspberry powders with activating function Tanh. Tanh function: Tanh squashes the real-valued number into the range [–1, 1]. The output is zero-centered.

As part of comparing the effectiveness of detecting the degree of saccharification and polysaccharide, the second research variant required the preparation of convolutional neural networks. The training set consisted of 1053 training cases (each as a 256 × 256 bitmap). The CNN based on network architecture MobileNet [21] was used in this variant. The MobileNet structure is explained by convolutional layer Depthwise Separable Convolution, which is based on using two sublayers: depthwise convolution and pointwise convolution. Depthwise convolution does one convolutional filter per each neuron in the input layer, and the pointwise convolution sublayer creates linear combinations for neurons in the output layer. MobileNet uses non-linearity both with batch normalization and for ReLu [21,34]. The structure of the devised CNN comprised the following:

- Input layer (InputLayer) i.e., a 256 × 256 × 1 bitmap with values of linear calibration between 0 and 1 (with discreet values among which, each two neighboring elements lie on the scale in the distance of 1/256). This is the initial tensor, which was sent to the first hidden layer;
- One standard convolutional layer (Conv2D), for each loaded image, 32 filters were used;
- Thirteen depthwise layers in separable convolution, where the depth of tensors (number of filters), depending on the number of layers, was 32, 64, 128, 256, or 1024. Each convolutional layer of this type consisted of sublayers in the given order:
  ○ Normalization (BatchNormalization), whose aim is to accelerate and increase the stability of artificial neural networks via the normalization of input layers via new centering or new calibration [35];
  ○ Activation (activation function ReLu) [17];
  ○ Depthwise convolution 2D;
  ○ Activation (activation function ReLu);
  ○ Pointwise convolution 2D;
  ○ Normalization (BatchNormalization);
- Sample operation global_average_pooling2d (unlike max_pooling used in standard convolutional layers). During global average joining, the size of the pool is still set at the size of the input data layer, but instead of maximum size, an average from the pool is taken into consideration. The aim of this action in building the model was to reduce the number of data transferred to fully-connected or densely-connected layers in the classifier;
- Operation dropout parameter set at 0.001. The dropout technique depends on the random selection of the determined number of characteristics in the input layer and on replacing them with zeros [36];
- Nine neurons in the output layer with activation function “softmax”. The activation function that was used is a mathematical function, with the help of which, the vector of numbers is transformed into the vector of probabilities. As a result of probability, each value is proportional to the relative scale of each vector value.

Between convolutional values, operation ZeroPadding2D occurred, which depends on widening each of two-dimensional characteristics of maps with one line and one column. Next, operation DepthWiseConv2D occurred, as a result of which the previously added squares were deleted.

### 2.5. Fourier Transform Infrared Spectroscopy

The research was carried out at Poznań University of Life Sciences with a spectrophotometer of Perkin Elmer company (Waltham, MA, USA) equipped with an ATR device with diamond as the internal reflection element. Spectra of the research samples were registered in absorbance function within the range of wave number 3550–350 cm^−1^ with a resolution 0.9 cm^−1^.

### 2.6. Statistica

Statistical analysis was carried out with the ANOVA method for individual research classes; a Tukey test was used as well with a significance level of *p* = 0.05 [37]. In order to carry out statistical analysis, the authors used Statistica 13.3. As part of the research, Principal Component Analysis (PCA) [5] was carried out, which allowed summarizing the information included in the set with texture coefficients. An observation of changeability of descriptors with correlation was also made.

## 3. Results and Discussion

### 3.1. MLP Learning

**Figure 2:**
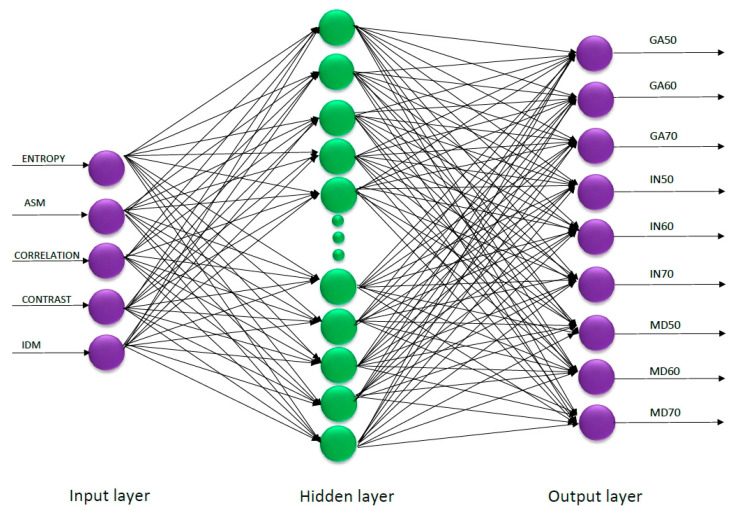
The structure of MLPN 5:5-24-9:1.

In order to determine adequate neural networks before the network training process, the minimal and maximum number of hidden layers was determined. For the minimal number of hidden layers in the neural model, the authors determined 10 layers, and the maximum number of hidden layers included 25 layers. It was noticed that for the training set used in the research characterized by a low number of hidden layers, neural networks obtained low classification efficiency. In case of exceeding 25 hidden layers, the networks had a tendency to overtrain, resulting in output with high error [38]. Among the networks that achieved high classification effectiveness with low RMSE were the networks that contained neurons in the hidden layer with activation function: logistic, tanh, exponential, and sigmoidal. The process of training with neurons in the hidden layer with the linear activation function obtained the highest RMSE. A simulation of networks was made, from which an adequate neural model was selected and saved. The selected neutral model was the one with the highest classification capabilities, which was created from 5 neurons in the input layer, 24 neurons in the hidden layer, and 9 neurons in the output layer (Figure 2). The MLP network was obtained with the activation function Logistic (for the hidden layers) and Softmax (for the output layer). The effectiveness of MLPN 5:5-24-9:1 on account of classification accuracy reached 0.962, while the RMSE value was at the level of 0.029 for the training, testing, and validation sets (Table 2). As part of the previous research with a digital camera and scanning microscope, the structure of microparticles in strawberry fruit powders was determined on the basis of parameters such as color, RGB, shape, and texture [7]. The MLP 30:30-19-4:1 network achieved a high classification effectiveness at the level of 0.99 and RMSE at the level of 0.03 with parameters related to each other such as color, RGB, and selected parameters of texture with 1960 training cases [7]. In the current research, an attempt was made to evaluate image texture with the structure of microparticles from a microscopic image. It was observed that in relation to the current research, the obtained MLP 5:5-24-9:1 (Figure 2), for which five texture descriptors were determined, was much more efficient in the classification of samples of fruit powders with a lower number of training cases (430 training cases).

In case of using coefficients of shape in the previous research, the effectiveness of classification was slightly lower and reached the value for MLP 9:9-9-4:1 at the level of 0.94, while RMSE was 0.13 (144 training cases) [7]. In the current research on the raspberry powders, the reason for the lower effectiveness of recognition of microparticles with shape is the number of training sets. In the MLP 8:8-44-9:1 network, for which 2903 cases in the form of microparticles of the raspberry powder were obtained, the lowest RMSE error was at the level of 0.01. Along with the increase in the number of cases in the form of microparticles, the effectiveness of recognition of those microparticles in the MLP network also increased [4].

In the current research, during the process of machine learning of networks, the Broyden–Fletcher–Goldfarb–Shanno (BFGS) algorithm was used, which informs about the curve of effectiveness function and allows determining the direction of searching for a minimum destination point with effectiveness within the network [39]. This algorithm with reference to the remaining methods seems to be more precise and more effective in the testing set. It results from the fact of taking into consideration a lower number of iterations in the process of training the network [39]. It concerns a comparison of effectiveness between previous research, for which BP algorithms were used, so training functions with a reverse propagation algorithm and compressed gradient function CG. For comparison, the BP and CG algorithms indicated that together with the increase in the number of iterations on the testing set, the effectiveness of identification of fruit powders obtained from chokeberry [12] or strawberry [7] also increased. In the recent research, for which the research material was raspberry powders, the level of iterations was also higher [4] than in the case of using the current method of trying, for which the value of iteration was 65.

### 3.2. CNN Learning

Table 2 shows the results of training convolutional networks. Network simulation was based on the Anaconda environment and packages for deep learning, i.e., TensorFlow and Keras. The TensorFlow package allows a substantial shortening of calculating time related to training artificial neural networks, especially related to image processing, thanks to parallel calculations with the GPU set [10,40]. The functions and methods of the Keras package allow efficient preparation of input data for the neural network [10,17]. As a result of the learning process in 214 iterations, an adequate convolution network was acquired, which was characterized by the highest classification capability. The CNN structure consisted of the input layer, i.e., as it was mentioned before it consisted of bitmaps with a resolution of 256 × 256 × 1, 1 convolutional layer, 13 separable convolution layers, and and 1 output layer determining 9 neural raspberry powders assigned to individual classes. An adaptive algorithm called “Adam” was used as an optimizer [41]. The “Relu” function was used as an activation function of the first and the hidden layers. CNN effectiveness was obtained at the level of 0.969, and the RMSE value was 0.071. Table 3 shows a fragment of architecture and the precise configuration of CNN called “mobilenet_1.00_256”.

As part of comparing the way of recognizing fruit powders including raspberry powders, the technique of deep learning was applied. As opposed to the previously used solutions with machine learning, the MLPN type of network turned out to be more effective than RBFN [7]. Comparing MLPN with CNN, one should understand the differences between machine learning and deep learning. Machine learning is a subset of Artificial Intelligence (AI) as an approach to try and achieve AI through systems that can find patterns in a set of data. Deep learning is one of the techniques for implementing machine learning. The current interest in this deep learning results from the growing popularity of cognitive data processing, which allows applications to understand the so-called human input signals and respond in a human-readable form. Deep learning technology has greatly improved the ability of computers to classify, recognize, detect, and describe data.

MLPN in machine learning depends on mapping the input variables (image, acoustic signal, or numeric data) of the input layer into the output variables of the output layer. In case of deep learning, neural networks carry out operation of mapping with data transformation starting from the input layer through other sublayers, ending at the output layer. In CNN, the convolution uses the kernel function and is connected with an increase in the linearity of tensor copies, on the basis of which operations are carried out, but it does not need to be connected with the increase of its measurement [40]. An example of using a convolution network is an algorithm of image classification on the account of the occurrence of shapes in an image that are repeatable, recognizable, and similar to each other. In such a case, the shape of the input tensor of the network can be described with shape (samples, image height, image width, depth as number of measurements of color space RGB) [40].

### 3.3. FTIR S Pectroscopy

**Figure 3:**
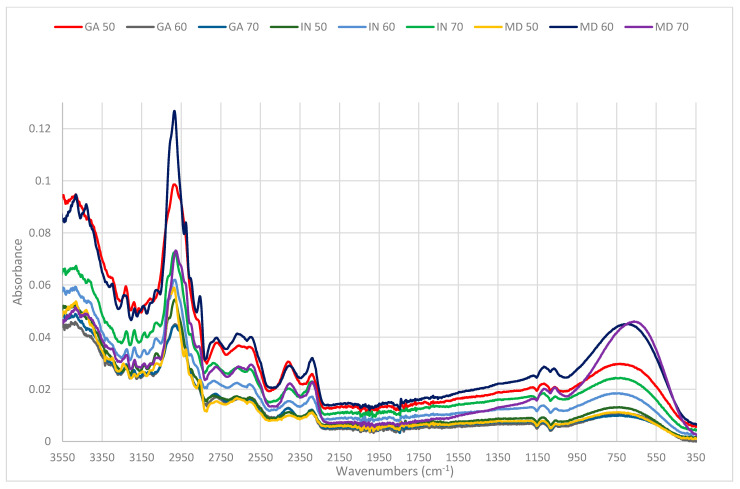
Spectrum in absorbance function for nine research trails.

In order to compare changes in the chemical structure of the samples of concentrate from raspberries with different carriers, infrared spectra were measured (Figure 3).

Analyzing the first spectral ranges of wave numbers from 2500 to 3550 cm^−1^ shows a wide asymmetric bond caused by valence vibrations of hydroxyl groups of bonds expanding the OH engaged in hydrogen bonds and expanding the vibration C-H. In this area, there occurs a sharp band with the wave number 2950 cm^−1^, which is assigned to the valance vibration of C-H asymmetric stretching of CH^2^ [42]. The highest intensity of this band can be observed for the trial with a 60% raspberry content and with a 40% maltodextrin carrier and for the trial with a 50% raspberry content and a 50% gum arabic carrier.

Other spectral regions between 1500 and 900 cm^−1^ are represented by strong expanding bonds C-O, C-C, C-O-H, and C-O-C, of different oligo and polysaccharides [43]. In this area, one can observe that the highest intensity of absorption is assigned to the band with 1085 cm^-1^. Intensity in this spectral region is assigned to the expanding vibrations of C-O, which indicate the highest intensity of absorption in the samples with maltodextrin.

The last spectral region “fingerprint” [44] below 900 cm^−1^ is responsible for crystal areas and indicates conformational changes in the material under research [45]. The region of the fingerprint is the region of infrared spectra where each organic compound has its own unique line of absorption. Those lines deliver information regarding the presence of various functional groups, which occur in the sample under research [44].

The analyzed samples coincide with the wave number of 700 cm^−1^, and the intensity of the absorption band is dependent on the proportion of ingredients of a given sample [46]. In this area, we can observe a delicate shift to maximum of absorption to shorter wave numbers for samples with a higher content of raspberries at the levels of 60% and 70% and with maltodextrin as carrier at the levels of 40% and 30%, respectively.

### 3.4. Statistical Analysis

In the next stage, variation analysis (ANOVA) was carried out, which allowed comparing nine research classes on the basis of texture descriptors (indirectly determined from microscopic images) and on the basis of the spectrum range (obtained via spectrophotometry in infrared). Table 4 presents the results of nine research classes different from each other in terms of the degree saccharification and the degree of polysaccharides.

While discussing entropy variables [15], which in the literature determine the amount of energy lost during physical reaction [47], one can observe similarity between research classes. It turns out that the highest similarity for the entropy variables occurred between research classes with a 30% content of sugar between IN and MD. Drawing a statistical comparison between research groups on the basis of entropy descriptor allows observing similarity in the group of raspberry powders with IN (IN50, IN60, IN70). Apart from it, the entropy variable was characterized by similarity in groups, among other things, for GA60, GA70, MD50, and MD60. In case of variables’ contrast and correlation, the analysis of variances showed that individual groups are different from each other. It was observed that the ASM variable, which is calculated as a second angular moment (the measurement of angular acceleration), was characterized by the homogeneity of variables (all variables are virtually equal to each other). In case of the IDM variable, it was observed that the most significant research groups are raspberry powders with a 50% content of sugar in the form of gum arabic. Statistical similarity between groups was observed for raspberry powders with sugar in the form of gum arabic (GA50, GA60, GA70).

As part of comparison with texture descriptors, the analysis for absorbance was carried out (FTIR). It was found, similarly to the IDM descriptor, that in the spectra (on the basis of FTIR), for the research classes of the raspberry concentrate with various carriers, the highest similarity occurs with gum arabic and maltodextrin (Table 4).

As part of the comparison of groups with hydroxyl bonds expanding OH, engaged in hydrogen bonds and expanding vibrations C-H, for which the highest intensity of bands with the research class GA50 was observed in the image, it can be statistically confirmed that with reference to the results of the research, it is a significant group (Table 4). In the current research that was carried out, it was observed that the biggest differences in terms of moistness and water activity occur in the research class GA50 [4].

In case of another area, which concerns oligo compounds and polysaccharides, it was observed that the highest intensity occurs with maltodextrin. In the analysis of variables, it was demonstrated that variables such as entropy correlation and contrast statistically determine that the significant groups are raspberry powders with various content of sugar in the form of maltodextrin.

**Figure 4:**
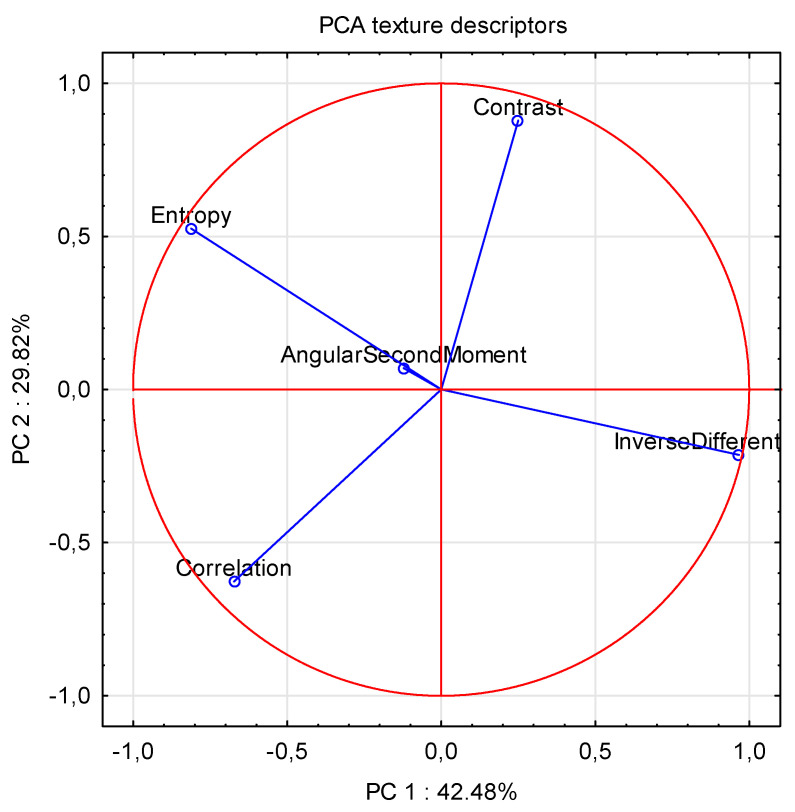
PCA of texture variables.

In the final stage Principal Component Analysis (PCA) was carried out. Data acquired from the learning set were pictured (texture descriptors). Figure 4 and Table 5 show that the entropy variables strongly and negatively correlated (R^2^ = −0.89) with the IDM variables. PCA analysis showed that the lowest correlation between texture descriptors occurred with the ASM variable.

Future research will concentrate on using artificial neural networks and image analysis in order to control the process of creating microparticles of fruit powders “online”. However, there is still a lack of knowledge about the basics of the process of creating microparticles. In the literature, one can find plenty of information about the build and morphology of the microparticles [48]. Unfortunately, there is no one determined method, which would allow designing a determined type of microparticles’ morphology. The authors are striving to acquire optimal solutions, which will allow monitoring the morphology of microparticles.

Summing up, monitoring the quality and parameters in the process of drying fruit powders (including the degree of saccharification and the type of polysaccharide) on the basis of modern techniques such as computer image analysis and neural modeling can lead to a substantial civilizational development. An increase in the repeatability of the obtained powders together with lowering costs of quality control can lower the prices of natural fruit powders, which can translate into their popularity among food producers, and this in turn can lead to an increased consumption of natural products among societies, eliminating in this way (at least to some degree) the use of synthetic additives.

## 4. Conclusions

The article presents nine different research classes (raspberry powders), which differ from each other in terms of the degree of saccharification and the type of polysaccharide. MLP was devised as being capable of quick and non-invasive identification of raspberry powders on the basis of the texture descriptors obtained (with SEM) in the process of low-temperature spray drying. In order to draw a comparison, an innovative solution with CNN was used, which similarly to MLPN was characterized by high classification effectiveness at the level of 0.96. Therefore, the model of deep artificial neural networks constitutes a valuable method of classification of the research classes of raspberry powders on the basis of their characteristics expressed in the form of a bitmap (image pixel). CNN compared with MLPN is a relatively low-cost solution in terms of calculating processes. In the future, it will translate into shortening the time of identifying research tasks online.

The analysis of variables allowed grouping the research classes in an effective way. PCA analysis allowed determining the influence of texture variables on the basis of correlations between them. The FTIR method allowed determining the research classes in a fast, non-invasive, and effective way (indirectly by determining the amount and the type of sugar).

## Figures and Tables

**Table 1:** Names of experimental variants used in the research task.

Name Research of Class	Type of Carrier	Ratio of Carrier
GA50	Gum Arabic	50%
GA60	Gum Arabic	40%
GA70	Gum Arabic	30%
IN50	Inulin	50%
IN60	Inulin	40%
IN70	Inulin	30%
MD50	Maltodextrin	50%
MD60	Maltodextrin	40%
MD70	Maltodextrin	30%

**Table 2:** The results of training networks.

Model ANN	MLPN	CNN
Training error	0.033	0.015
Validation error	0.016	0.086
Testing error	0.047	0.093
Quality of learning	0.967	0.998
Quality of validation	0.953	0.956
Quality of testing	0.984	0.952
Learning cases	430	1023
Training algorithm	BFGS 65	Adam
Accuracy	0.962	0.969
RMSE	0.029	0.065

**Table 3:** Configuration of the proposed CNN architecture. The total number of model parameters is 3,237,513.

Layer (Type)	Output Shape	Param #
Input_tensor (InputLayer)	(None, 256, 256, 1)	0
conv1_pad (ZeroPadding 2D)	(None, 256, 256, 1)	0
conv1 (Conv2D)	(None, 128, 128, 32)	288
conv1_bn (BatchNormalization)	(None, 128, 128, 32)	128
conv1_relu (Relu)	(None, 128, 128, 32)	0
conv1_dw_1 (DeptwiseConv2D)	(None, 128, 128, 32)	288
conv1_bn (BatchNormalization)	(None, 128, 128, 32)	128
conv1_relu (Relu)	(None, 128, 128, 32)	0
conv_pw_1 (Con2D)	(None, 128, 128, 64)	2048
conv_pw_bn (BatchNormalization)	(None, 128, 128, 64)	256
conv_pw_relu (Relu)	(None, 128, 128, 64)	0
conv_pad_2 (ZeroPadding 2D)	(None, 129, 129, 64)	0
conv_dw_1 (DeptwiseConv2D)	(None, 64, 64, 64)	576
conv_dw_2_bn (BatchNormalization)	(None, 64, 64, 64)	256
conv_dw_2relu_ (Relu)	(None, 64, 64, 64)	0
conv_pw_2	(None, 64, 64, 128)	8192
⋮	⋮	⋮
conv_dw_13 (DeptwiseConv2D)	(None, 8, 8, 1024)	9216
conv_dw_13_bn (BatchNormalization)	(None, 8, 8, 1024)	4096
conv_dw_13_relu (ReLU)	(None, 8, 8, 1024)	0
conv_pw_13 (Conv2D)	(None, 8, 8, 1024)	1,048,576
conv_pw_13_bn (BatchNormalization)	(None, 8, 8, 1024)	4096
conv_pw_13_relu (ReLU)	(None, 8, 8, 1024)	0

**Table 4:** Texture analysis and FTIR of raspberry powders containing 50, 60, and 70% of raspberry concentrate solids obtained with gum arabic (GA), inulin (IN), and maltodextrin (MD) by dehumidified air-assisted spray drying.

Name Research of Class	Entropy		Contrast		Correlation	
GA50	9.44741 ± 0.07429	e	411.17285 ± 49.22679	f	0.00019 ± 0.00003	e
GA60	9.25423 ± 0.12064	d	363.11773 ± 50.70518	e	0.00016 ± 0.00002	bc
GA70	9.27491 ± 0.07331	d	325.60968 ± 32.00526	d	0.00018 ± 0.00002	de
IN50	9.16783 ± 0.11597	bc	278.80630 ± 24.96329	c	0.00016 ± 0.00002	bcd
IN60	9.10750 ± 0.13881	ab	306.03450 ± 28.23464	cd	0.00017 ± 0.00002	cd
IN70	9.07736 ± 0.11930	a	240.10396 ± 18.03900	b	0.00018 ± 0.00001	de
MD50	9.19857 ± 0.15313	cd	619.43057 ± 82.97847	g	0.00014 ± 0.00001	a
MD60	9.19922 ± 0.09826	cd	212.04252 ± 30.00097	a	0.00028 ± 0.00006	f
MD70	9.04692 ± 0.14894	a	325.23585 ± 30.58993	d	0.00015 ± 0.00001	ab
**Name Research of Class**	**ASM**		**IDM**		**FTIR**	
GA50	0.00099 ± 0.00065	a	0.11793 ± 0.00879	a	0.017993 ± 0.019006	a
GA60	0.40517 ± 1.86809	b	0.13977 ± 0.01456	b	0.009461 ± 0.014758	b
GA70	0.00090 ± 0.00103	a	0.13950 ± 0.00914	b	0.012608 ± 0.014599	c
IN50	0.00241 ± 0.00172	a	0.15096 ± 0.01822	c	0.014077 ± 0.016693	c
IN60	0.00301 ± 0.00197	a	0.15454 ± 0.01935	cd	0.014508 ± 0.016117	d
IN70	0.00224 ± 0.00163	a	0.16120 ± 0.01597	de	0.018740 ± 0.016393	e
MD50	0.00470 ± 0.00199	a	0.14930 ± 0.01573	bc	0.023208 ± 0.019160	bc
MD60	0.00016 ± 0.00001	a	0.11830 ± 0.00523	a	0.013685 ± 0.017191	f
MD70	0.00372 ± 0.00202	a	0.16977 ± 0.02016	e	0.033704 ± 0.025317	e

a–g: the differences between mean values with the same letter in columns were statistically insignificant (*p* < 0.05).

**Table 5:** The results of correlation of texture variables.

Desrciptor	ASM	Contrast	Correlation	IDM	Entropy
ASM	1.000000				
Contrast	0.011270	1.000000			
Correlation	0.041610	−0.560466	1.000000		
IDM	−0.053445	0.041667	−0.521530	1.000000	
Entropy	0.074450	0.167412	0.132865	−0.885284	1.000000

## Data Availability

Not applicable.
